# An age-dependent reversal in the protective capacities of JNK signaling shortens *Caenorhabditis elegans* lifespan

**DOI:** 10.1111/j.1474-9726.2012.00829.x

**Published:** 2012-08

**Authors:** Kwame Twumasi-Boateng, Tim W Wang, Linda Tsai, Kuang-Hui Lee, Ali Salehpour, Sudarshan Bhat, Man-Wah Tan, Michael Shapira

**Affiliations:** 1 Graduate Group in Microbiology, University of California at BerkeleyCA 94720-3102, USA; 2 Department of Integrative Biology, University of California at BerkeleyCA 94720-3102, USA; 3 Departments of Genetics and Microbiology and Immunology, Stanford University School of MedicineStanford, CA 94305, USA

**Keywords:** stress, *C. elegans*, JNK, KGB-1, antagonistic pleiotropy, lifespan, stress

## Abstract

Stress-activated protein kinase (SAPK) pathways are evolutionarily conserved signaling modules that orchestrate protective responses to adverse environmental conditions. However, under certain conditions, their activation can be deleterious. Thus, activation of the c-Jun N-terminal kinase (JNK) SAPK pathway exacerbates a diverse set of pathologies, many of which are typical of old age. The contexts determining whether the outcome of JNK signaling is protective or detrimental are not fully understood. Here, we show that the age of an animal defines such a context. The *Caenorhabditis elegans* JNK homolog, KGB-1, provides protection from heavy metals and protein folding stress in developing animals. However, we found that with the onset of adulthood, KGB-1 activity becomes detrimental, reducing stress resistance and lifespan. Genetic analyses coupled with fluorescent imaging linked this phenotypic switch to age-dependent antagonistic modulation of DAF-16/FOXO: KGB-1 activation enhanced DAF-16 nuclear localization and transcriptional activity during development but decreased it in adults. Epistasis analyses showed that DAF-16 was necessary and sufficient to explain some of the *kgb-1*-dependent detrimental phenotypes, but not all. The identification of early adulthood as a point following which the contribution of KGB-1 activity reverses from beneficial to detrimental sheds new light on the involvement of JNK signaling in age-related pathologies. Furthermore, the age-dependent reversal has intriguing implications for our understanding of aging.

## Introduction

Dealing with adverse environmental perturbations is a fundamental necessity for all organisms. It is therefore not surprising that stress-activated protein kinase pathways are among the most ancient and conserved signaling modules in metazoa. The two archetypical pathways comprising this category are the p38 and the c-Jun N-terminal kinase (JNK). They are activated by a range of adverse environmental conditions, including protein folding stress, oxidative stress, and infection, and function in a typical MAPK three-tier, scaffold-protein-aided hierarchy, in which a mitogen-activated protein (MAP) kinase kinase kinase phosphorylates and activates an MAP kinase kinase, which activates either the p38 or the JNK MAP kinases, which modify the activity of numerous and diverse targets through phosphorylation (Kyriakis & Avruch, 2001; Morrison & Davis, 2003).

JNK proteins were shown in mammals to respond to various stress conditions, as well as to inflammatory cytokines, and to modify proteins as diverse as ubiquitin ligases, microtubule-associated proteins, and several transcription factors (reviewed in (Weston & Davis, 2007)). In their protective capacity, JNK proteins contribute to microtubule stabilization, neurite growth, autophagy, and induction of cytoprotective gene expression (Chang *et al.*, 2003; Hayakawa *et al.*, 2003; Ogata *et al.*, 2006; Podkowa *et al.*, 2010). JNK signaling also plays pivotal roles in development, as demonstrated by embryonic lethality in mice lacking both of the two ubiquitously expressed isoforms JNK1 and JNK2 (in contrast to mice lacking the neuronal-specific JNK3, which are viable) (Kuan *et al.*, 1999). In contrast to these beneficial contributions, JNK signaling has also been shown to have detrimental consequences. JNK activation can be pro-apoptotic and is associated with the exacerbation of a diverse set of pathologies, many of which are typical of old age. These include Alzheimer's disease, ischemia/reperfusion-induced tissue damage in the heart and brain, and insulin resistance (Morishima *et al.*, 2001; Kuan *et al.*, 2003; Baines & Molkentin, 2005; Sabio *et al.*, 2010). Accordingly, *jnk3* disruption protects mice from ischemia-associated damage, and *jnk1* disruption improves insulin sensitivity in a mouse model of obesity (Hirosumi *et al.*, 2002; Kuan *et al.*, 2003). What determines the outcome of JNK activation is not fully known, but a recurrent theme is that the mode/context of activation plays an important role. It has been shown that interactions with other signaling pathways can modulate the activation, and/or contribution, of JNK signaling (Lamb *et al.*, 2003; Kamata *et al.*, 2005); others have suggested that the duration of activation determined the outcome of JNK signaling – short activation leading to protection, a prolonged one leading to cell death (Ventura *et al.*, 2006; Wei *et al.*, 2011); yet another hypothesis, referring to the whole organism, suggested that JNK activation may have different outcomes depending on tissue specificity of this activation (Sabio *et al.*, 2010).

Invertebrate model organisms such as *Drosophila melanogaster* and *Caenorhabditis elegans* are useful for understanding the effects and interactions of JNK proteins, in particular by allowing dissection of cytoprotective gene expression and analyzing tissue-specific contributions (Biteau *et al.*, 2011). Work in these organisms also enabled studying the interactions between the JNK pathway and insulin/insulin-like-growth-factor (IGF) signaling (IIS), arguably the most significant mechanism of aging regulation. IIS plays a pivotal role in coordinating metabolic homeostasis and determining longevity in a variety of organisms (Tatar *et al.*, 2003). Best characterized in *C. elegans,* the main contribution of IIS is in antagonizing nuclear localization of the DAF-16/FOXO transcription factor, which controls the expression of a robust stress-protective transcriptional program (Murphy *et al.*, 2003). Thus, by restricting nuclear localization of DAF-16, IIS limits resistance to oxidative stress and infection and ultimately reduces lifespan (Henderson & Johnson, 2001; Garsin *et al.*, 2003). JNK proteins were shown to phosphorylate several central and auxiliary components of the IIS pathway (reviewed in (Biteau *et al.*, 2011). The outcome of these interactions is typically the promotion of FOXO nuclear localization and an increase in stress resistance and lifespan (Oh *et al.*, 2005; Wang *et al.*, 2005). However, similar to mammals, JNK activation may also have detrimental consequences in invertebrates. In drosophila, uncontrolled JNK activity impairs epithelial gut integrity (Seisenbacher *et al.*, 2011) and causes improper differentiation in the aging intestine (Biteau *et al.*, 2008). Also, flies heterozygous for the disruption of a dual-specificity phosphatase, which negatively regulates JNK signaling, show increased oxidative stress resistance and lifespan, but homozygotes are developmentally lethal (Martin-Blanco *et al.*, 1998; Wang *et al.*, 2003). Similarly, loss of the orthologous *C. elegans* phosphatase, VHP-1, results in developmental lethality that is rescued by the disruption of the cognate JNK homolog KGB-1 (Mizuno *et al.*, 2004). Thus, in invertebrates, as in vertebrates, JNK activation can have contrasting outcomes. However, as in mammals, what determines these outcomes is not fully understood.

**Fig. 1:**
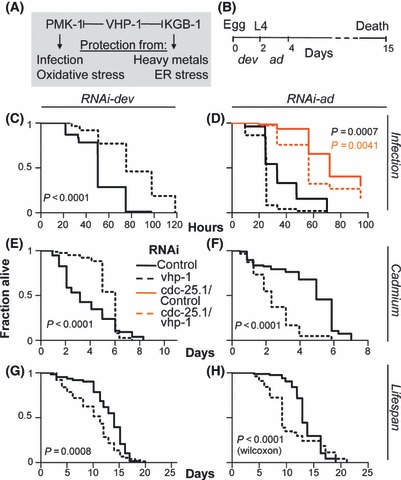
An age-associated reversal in the vhp-1 RNAi phenotype (A) VHP-1 negatively regulates both PMK-1 and KGB-1. (B) Timeline for the life of wild-type *Caenorhabditis elegans* at 25 °C. (C–H) Survival curves for wild-type animals fed with RNAi-expressing *Escherichia coli*, as designated, and subsequently exposed to *Pseudomonas aeruginosa* (C, D), 100 μm cadmium (E, F), or dead food bacteria (G, H). Knock-down was performed during development (dev = egg until L4 stage) (C, E, G), or early adulthood (ad = L4 stage for 2 days), (D, F, H); or, following sterilization achieved by a development-stage exposure to cdc-25.1 RNAi [orange curves in (D)]. Graphs are representatives of ≥ 2 experiments showing similar results.

Using *C. elegans*, we show that age defines a context determining the outcome of JNK activation. *Caenorhabditis elegans* has three JNK homologs, similar to mammals. The neuronal JNK-1 provides protection from oxidative and heat stress and interacts with the IIS pathway leading to nuclear localization of DAF-16 (Oh *et al.*, 2005; Neumann-Haefelin *et al.*, 2008). Of the other two JNK homologs, KGB-2 is uncharacterized, and KGB-1 was shown to be necessary both for germline proliferation and for protection from heavy metals and protein folding stress (Smith *et al.*, 2002; Mizuno *et al.*, 2004, 2008). Both KGB-1 and the p38 homolog PMK-1, which provides protection from infection and oxidative stress (Kim *et al.*, 2002; Inoue *et al.*, 2005), are negatively regulated by the dual-specificity phosphatase VHP-1, a ubiquitously expressed MKP-7 homolog (Fig. 1A). Previous work showed that knocking down the expression of *vhp-1* during larval development increased phosphorylation of PMK-1 and, downstream to it, resistance of worms to infection with the bacterial pathogen *Pseudomonas aeruginosa* (Kim *et al.*, 2004). However, we found that knocking down *vhp-1* past development had the opposite effect – decreased resistance to infection – which depended on *kgb-1*. We show that the contribution of *kgb-1* to stress resistance reverses with age – from a protective role in dealing with heavy metals and protein folding stress in developing larvae to being generally detrimental in adults, causing a decrease in resistance to heavy metals and protein folding stress, in addition to infection resistance, and shortening lifespan under normal conditions. The age-dependent switch in KGB-1’s function was linked to age-dependent antagonistic modulation of DAF-16 – promoting DAF-16 activity during development, but attenuating it in adults. Our results demonstrate that age can be a context determining the outcome of JNK activation and describe a molecular mechanism underlying this phenomenon.

## Results

### Age reverses the protective effect of *vhp-1* knock-down

Our original intention was to study the contribution of PMK-1 activation to infection resistance. By knocking down the expression of *vhp-1* during the 2 days of larval development (RNAi-dev, Fig. 1B), we increased the resistance of worms to infection, as previously reported (Fig. 1C) (Kim *et al.*, 2004). However, to our surprise, the same knock-down in young adult animals, only 2 days older (RNAi-ad), caused the opposite effect, rendering worms more susceptible to infection (Fig. 1D). Thus, a 2-day difference in the onset of RNAi treatment reversed the consequences of *vhp-1* knock-down from a median of 55% increase in infection resistance to a 34% decrease. This observation led us to focus our efforts on trying to understand the mechanism underlying such a switch.

### The age-dependent reversal in the vhp-1 RNAi phenotype is independent of reproductive status or growth

Trade-offs between stress resistance and growth, or reproduction, are well documented (Harshman & Zera, 2007). Development of *vhp-1* mutants was previously reported to arrest at the L3 larval stage (Mizuno *et al.*, 2004), and *vhp-1(RNAi-dev)* animals showed a high percentage of small animals that produced either no or few progeny. While *vhp-1(RNAi-ad)* animals showed no gross defects in fertility, minor effects might still have existed. This raised the possibility that the reversal in the effect of vhp-1 RNAi might be an indirect consequence of age-dependent effects on growth and reproduction. To test this possibility, we examined wild-type animals rendered sterile prior to adult-stage *vhp-1* knock-down, by *cdc-25.1* knock-down. We found that these animals showed the same decreased infection resistance phenotype as fertile animals (Fig. 1D, orange lines). Furthermore, both increased infection resistance in *vhp-1(RNAi-dev)* animals and decreased resistance in *vhp-1(RNAi-ad)* animals were replicated in sterile *glp-4* and *spe-26* mutants, which lack gonads and sperm, respectively (Fig. S1).

Effects on animal size were also not the cause of the vhp-1 RNAi phenotype reversal: animals exposed to vhp-1 RNAi throughout development, plus the first 2 days of adulthood (4 days instead of two), showed stunted growth and reduced fecundity, similar to *vhp-1(RNAi-dev)* animals, but were less resistant to infection, similar to their age-matched *vhp-1(RNAi-ad)* animals (Fig. S2). Together, these experiments rule out the involvement of reproductive status, or size, in the age-associated reversal in the *vhp-1* knock-down infection resistance phenotype.

### The age-dependent phenotype reversal represents a more general shift in the ability to resist environmental stress

In addition to infection resistance, we found that an age-dependent phenotype reversal also appeared in *vhp-1(RNAi)* animals challenged with heavy metals. Cadmium resistance changed from a 40% increase in *vhp-1(RNAi-dev)* animals (Fig. 1E) to a 50% decrease in *vhp-1(RNAi-ad)* animals (Fig. 1F), compared to age-matched control-treated animals.

Because stress resistance is tightly linked to lifespan, we examined the effects of *vhp-1* knock-down on lifespan. The median lifespan of *vhp-1(RNAi-dev)* animals on dead *Escherichia coli* was 84% of that of age-matched control-treated animals (Fig. 1G); more prominently, median lifespan of *vhp-1(RNAi-ad)* animals was 69% of controls (Fig. 1H). Thus, the long-term consequences of *vhp-1* knock-down are detrimental, even in the absence of exogenous stress.

### *kgb-1* plays a central role in the age-dependent reversal in the effects of *vhp-1* knock-down

**Fig. 2:**
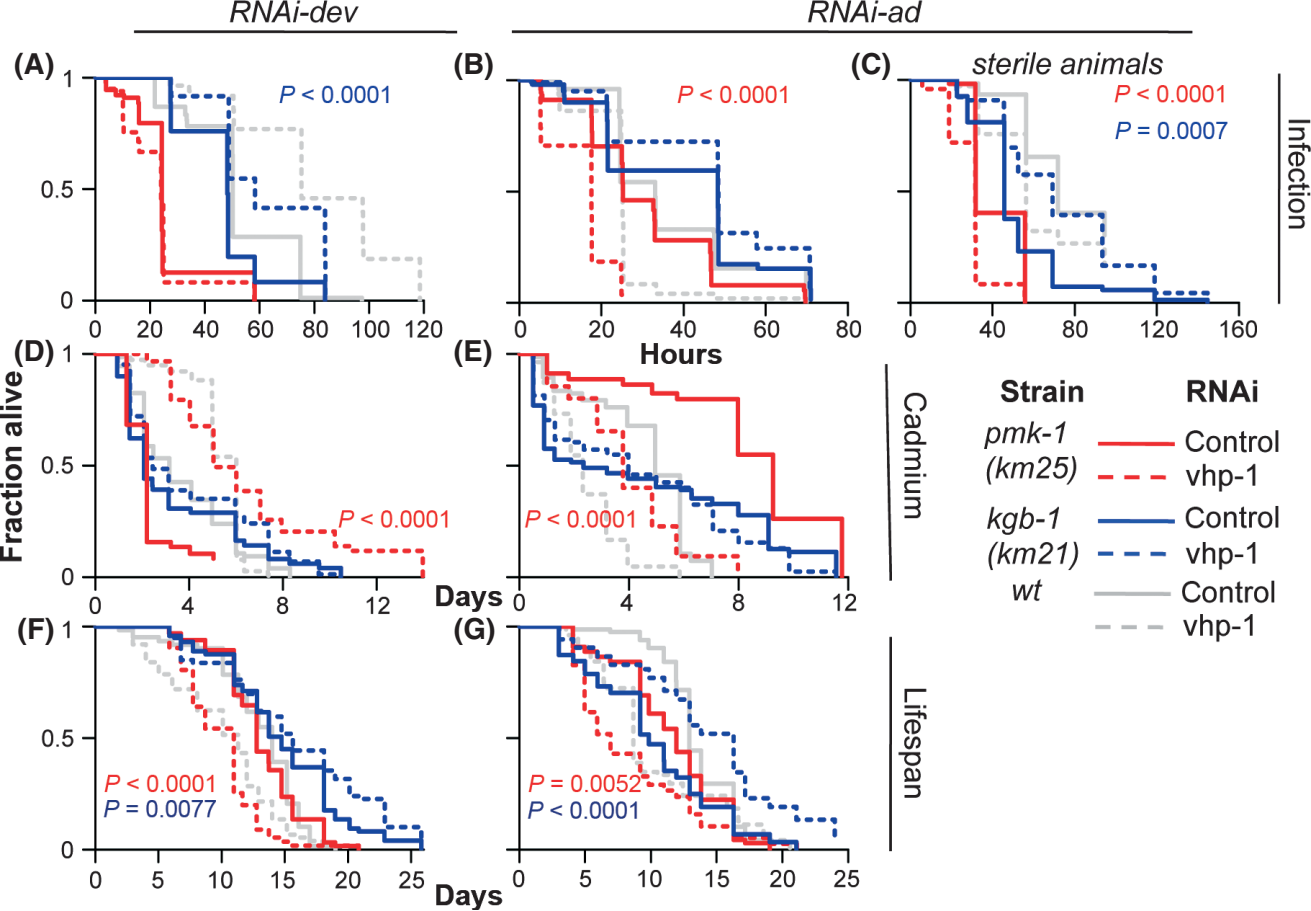
*kgb-1* plays a central role in the age-associated reversal in the vhp-1 RNAi phenotype. Survival curves for mutant animals fed with RNAi-expressing *Escherichia coli*, as designated, and subjected to conditions as in Fig. 1. Gray curves, representing survival of *w*ild-type animals, are the same as in Fig. 1 and are shown for comparison. Knock-down was performed during development (dev) (A, D, F), or early adulthood (ad), (B, C, E, G); in C, adult-stage *vhp-1* knock-down was performed following development-stage knock-down of *cdc-25.1*. Graphs are representatives of ≥ 2 experiments showing similar results. Curves without designated *P*-values are not significantly different (*P* > 0.05).

Ruling out dependence on growth and reproduction led us to hypothesize that the reversal in the *vhp-1* knock-down phenotype might have reflected age-associated changes in signaling by the two stress-activated protein kinases regulated by VHP-1. We therefore examined *pmk-1* and *kgb-1* mutants to identify which of the two genes was necessary for which of the age-dependent phenotypes. In agreement with previous reports, *pmk-1* disruption suppressed the increased infection resistance following development-stage *vhp-1* knock-down (Fig. 2A)(Kim *et al.*, 2004). On the other hand, increased cadmium resistance following the same RNAi treatment was suppressed instead by *kgb-1*-disruption (Fig. 2D). As for the detrimental effects of *vhp-1* knock-down in adults, all depended on *kgb-1* alone (Fig. 2B, C, E–G)(and in both fertile and sterile animals (Fig. 2B, C)). This was observed in animals carrying two different mutant *kgb-1* alleles, *km21* and *um3*, ruling out allele-specific effects (Fig. S3). On the other hand, mutants for the two other *C. elegans* JNK homologs, *jnk-1* and *kgb-2*, responded to *vhp-1* knock-down as wild-type animals, ruling out their involvement (not shown). Thus, a previously unknown detrimental contribution of *kgb-1* in adult animals was identified that contrasts with its early-life protective role against cadmium toxicity. Similarly, opposing contributions were also observed for the role of *kgb-1* in protection from protein folding stress caused by the N-glycosylation inhibitor tunicamycin (Fig. S4). Together, these results demonstrated that the reversal in the vhp-1 RNAi phenotype is *kgb-1*-dependent and further suggested a post-developmental switch in the contribution of *kgb-1*, from beneficial (for cadmium and tunicamycin protection) to generally detrimental.

### *kgb-1* knock-down corroborates age-dependent reversal in *kgb-1*’s contribution to cadmium resistance

**Fig. 3:**
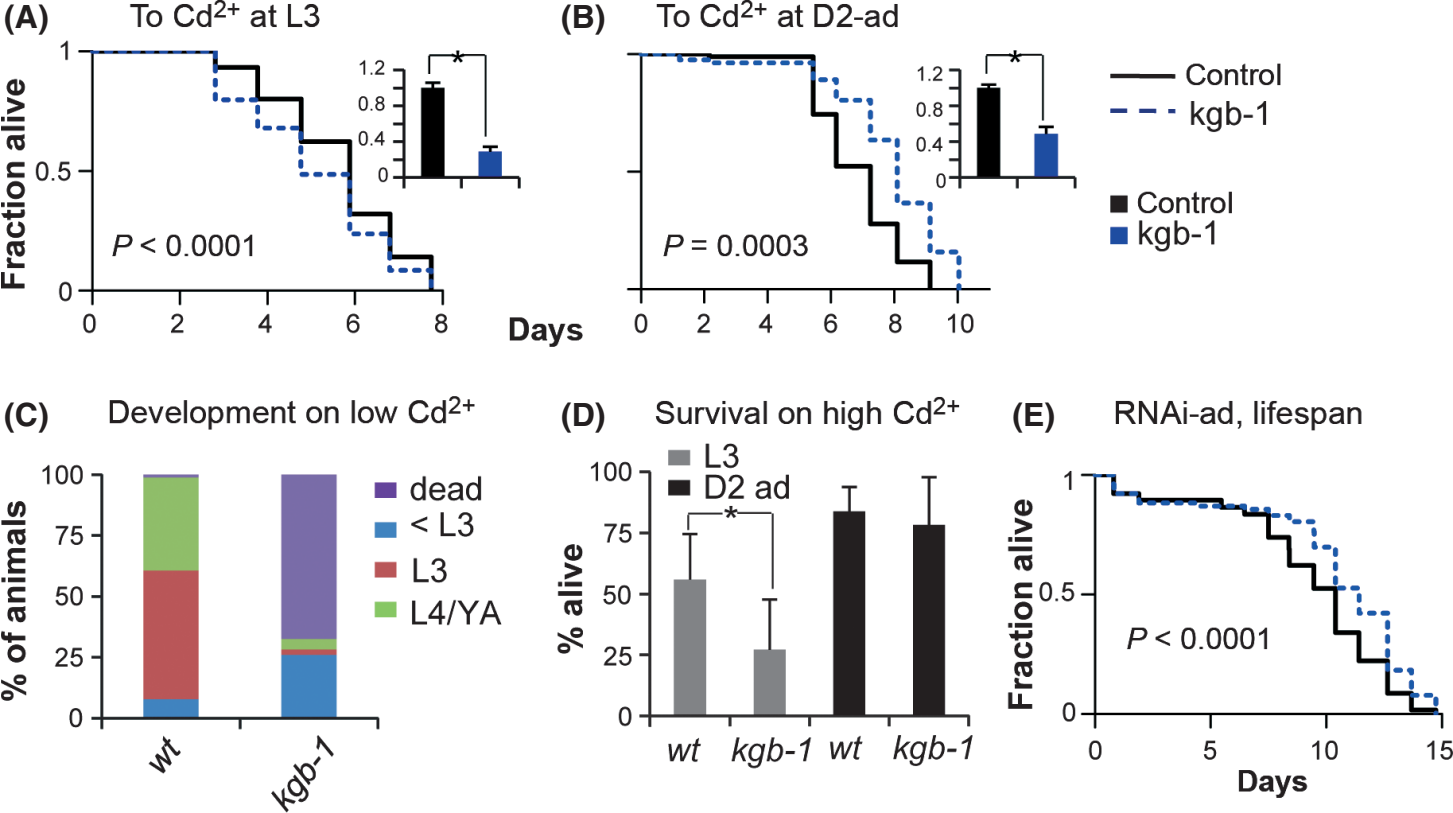
*kgb-1* protects larvae from cadmium but is detrimental in adults. (A, B) Survival curves for animals treated for two generations with kgb-1 RNAi and exposed to 100 μm CdCl_2_, as L3 larvae (A) or as 2-day old adults (B); shown are representatives of three experiments. Insets present qRT-PCR evaluation of the extent of *kgb-1* knock-down in the respective ages, presented as average ± SD (of two experiments) of fold change over control treatment: *P < 0.001. (C) Developmental status and survival of untreated wild-type and *kgb-1(km21)* worms following a 3-day exposure to 50 μm cadmium (at 25 °C). Shown is a representative of two independent experiments, each containing at least 50 worms per strain. (D) Survival following acute exposure to cadmium (5 mm, in liquid). Animals exposed to cadmium at the indicated stages were assayed for survival 10 h later (L3 larvae) or between 11 and 17 h later (D2 adults). Shown are means ± SD of two independent experiments, *P = 0.00260. *N* = 60 per strain per experiment. (E) *kgb-1* knock-down in wild-type adults (beginning at L4 for 2 days) extends lifespan. Shown is a representative of three experiments.

One observation that appears to conflict with the hypothesis that KGB-1’s function switches to detrimental in adulthood is that lifespan of *kgb-1* mutants is often shorter than that of wild-type animals (Fig. 2G). However, the discrepancy could be resolved if it is assumed that *kgb-1* had an early-life beneficial contribution that was greater than its late-life detrimental one. Supporting a bimodal contribution, we noticed that the survival of *kgb-1* mutants on cadmium showed an initial fast phase of dying, followed by the survival of the remaining animals (50% of the initial number) to a maximum lifespan that exceeded that of wild-type animals (Fig. 2E). To better resolve the effect of *kgb-1* disruption on stress resistance, we examined *kgb-1(RNAi)* animals, expecting that residual *kgb-1* may be sufficient to provide essential functions, yet would allow testing age-dependent contributions. When exposed to cadmium as larvae, animals treated with kgb-1 RNAi (beginning *in utero*) showed reduced survival compared to controls, supporting an early-life beneficial contribution (Fig. 3A). In contrast, *kgb-1(RNAi)* animals exposed to cadmium at day two of adulthood showed increased survival, supporting a late-life detrimental contribution (Fig. 3B). Quantitative RT-PCR measurements showed comparable knock-down of *kgb-1* expression in both ages, with ‘residual’ levels amounting to 30–50% of control levels (Fig. 3 insets and >Fig. S5). Together, these results supported the hypothesis of an age-dependent switch in *kgb-1*’s contribution to survival. Furthermore, by producing essentially the reverse image of the effects of *vhp-1* knock-down on cadmium resistance, the outcome of *kgb-1* knock-down demonstrated that the switch is a characteristic of the normal KGB-1 physiology and not merely a product of uncontrolled activation.

### Loss of *kgb-1*’s larval-protective capacities in adults

Our survival analyses followed the effects of the preceding age-dependent knock-down, which determined the state of animals at the beginning of the analysis. However, the longer the survival analysis, the more diluted the effects of the initial knock-down might be. To better dissect *kgb-1*’s age-dependent contributions, we employed *kgb-1* mutants and knock-down animals in assays limited to defined life phases. We began by comparing the development of *kgb-1* and wild-type animals. Development of the two strains was indistinguishable under normal conditions. However, when exposed to cadmium at the egg stage, wild-type larvae showed delayed development; nevertheless, within 3 days, around 40% reached adulthood (Fig. 3C). In contrast, the majority of *kgb-1* larvae exposed to cadmium died during development, with only 4% of total reaching adulthood. Thus, in larvae, *kgb-1* is essential for survival and development under the adverse conditions of cadmium exposure. Next, we evaluated the short-term contribution of *kgb-1* to stress resistance in larvae. This was performed in the context of an acute exposure to high cadmium concentration (5 mM) and was compared to that of 2-day-old adults. Within 10 h of exposure, 75% of *kgb-1* larvae were dead, compared with 45% among wild-type larvae, supporting a short-term protective role for KGB-1 in developing animals (Fig. 3D). In contrast, *kgb-1* disruption had no effect on cadmium resistance in adults, suggesting that in adults, the contribution of *kgb-1* to cadmium resistance was negligible. Finally, further focusing on *kgb-1*’s contribution in adults, we found that knocking down its expression only after development was completed resulted in lifespan extension (Fig. 3E). Thus, as the beneficial contribution of *kgb-1* to cadmium resistance wanes, it becomes a negative factor, compromising animal survival.

### Upstream activation of KGB-1 does not change with age

**Fig. 4:**
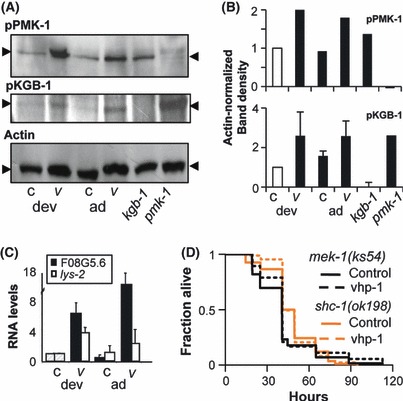
PMK-1 and KGB-1 activation following *vhp-1* knock-down does not change with age. (A) Immunoblot of wild-type or indicated mutant animals (negative controls) treated with control (c) or *vhp-1* (v) RNAi as designated. (B) Actin-normalized band densities in one experiment for pPMK-1 or means ± SD of four gels representing two experiments for pKGB-1 presented as fold changes over values in *control(RNAi-dev)* animals (empty columns). (C) RNA levels, measured by quantitative (q)RT-PCR, of PMK-1’s downstream targets *lys-2* and F08G5.6. Means ± SD of two independent experiments, presented as fold changes over values in striped columns. (D) Infection survival curves following adult-stage *(RNAi-ad)* treatment.

The age-associated changes in the outcome of KGB-1 activation could have been due to changes in events either upstream or downstream to KGB-1 activation. Because VHP-1 modulates phosphorylation of both KGB-1 and PMK-1, we examined whether changes occurred in the overall balance of this network. Basal RNA levels of *kgb-1*, *pmk-1*, and the *vhp-1*a isoform at day two of adulthood were higher than those in L4 larvae, while *vhp-1b* levels did not change with age (Fig. S6; data not shown). As the functional output of this network depends not simply on expression levels but on levels of the phosphorylated active proteins, we analyzed phosphorylation levels for both PMK-1 and KGB-1. Immunoblotting showed that basal levels of phosphorylated proteins did not differ significantly between worms of the two examined ages for either PMK-1 or KGB-1 (Fig. 4A, B). *vhp-1* knock-down increased the phosphorylation of both PMK-1 and KGB-1 irrespective of age, suggesting that both were similarly activated during development and adulthood (Fig. 4A, B). In addition, increases in PMK-1 phosphorylation were found to be associated with increased expression (also age-invariable) of two downstream targets of PMK-1, the infection-protective genes *lys-2* and F08G5.6 (Shapira *et al.*, 2006; Troemel *et al.*, 2006), suggesting that PMK-1 retained its infection-protective contribution in adults (Fig. 4C). Nevertheless, the constitutive beneficial contribution of PMK-1 appears to be superseded in adults by the overriding detrimental effects of KGB-1 (Fig. 1D) and becomes apparent only once *kgb-1* is disrupted (Figs 2B, C and S3B).

The MAPK kinase MEK-1 is the main activator of KGB-1 and requires the scaffold protein SHC-1 for this activation. Both were previously shown to be essential for KGB-1’s ability to provide protection from heavy metal stress during development (Mizuno *et al.*, 2008). Disruption of either *mek-1* or *shc-1* also suppressed the detrimental consequences of adult-stage *vhp-1* knock-down (Fig. 4D), demonstrating that the machinery required for the activation of KGB-1 is equally important for its development-stage beneficial and adult-stage detrimental contributions. Thus, the switch in *kgb-1*’s contribution cannot be explained by changes in KGB-1 activation and instead points to events downstream to this activation as those that change with age.

### Age-dependent modulation of DAF-16 downstream to KGB-1 activation

**Fig. 5:**
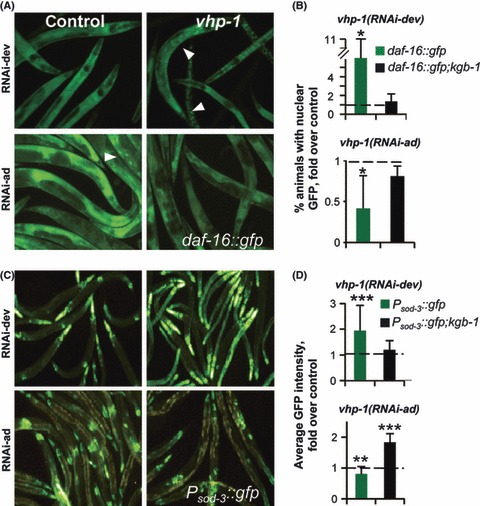
*kgb-1* contributes to age-dependent modulation of DAF-16 nuclear localization. (A) Representative images of DAF-16::GFP-expressing animals exposed to RNAi as designated; arrowheads mark localization in intestinal nuclei. (B) Quantification of DAF-16::GFP nuclear localization in images as in A, shown as the percentage of *vhp-1(RNAi)* animals with intestinal nuclear localization of DAF-16::GFP, and as fold changes over control-treated animals; Means ± SD of 4–5 experiments in wild-type animals, or three, in *kgb-1(km21)* mutants. *P < 0.05, **P < 0.0001. Basal prevalence of DAF-16 nuclear localization (in animals treated with control RNAi) was 0.6–11% in wild-type *RNAi-dev* animals, 0–6% in *kgb-1;RNAi-dev* animals, 14–23% in wild-type *RNAi-ad* animals and 2–9% in *kgb-1;RNAi-ad* animals. (C) Representative images, and their quantification (D) of GFP levels in *P*_*sod-3*_*::gfp* or *P*_*sod-3*_*::gfp;kgb-1(um3)* animals, shown as in B. Means ± SD of three experiments (*P*_*sod-3*_*::gfp*) or 2 experiments (*P*_*sod-3*_*::gfp;kgb-1*) with 20–30 animals per group; **P = 0.0007, ***P < 0.0001.

Given *kgb-1’s* contribution to lifespan determination and stress resistance, we wondered whether KGB-1 interacted with the IIS pathway. Insulin signaling restricts lifespan and stress resistance mainly by inhibiting nuclear localization of the conserved FOXO transcription factor DAF-16 (Henderson & Johnson, 2001). We therefore examined the effect of *vhp-1* knock-down on DAF-16. Using a transgenic strain expressing a functional GFP-tagged DAF-16 (Henderson & Johnson, 2001), we found that *vhp-1* knock-down in developing animals significantly increased DAF-16::GFP nuclear localization in intestinal cells (independently of its effects on development itself, see Fig. S7), while its knock-down in adults reduced it (Fig. 5A, B). Both the larval-stage increase in DAF-16 nuclear localization and the adult-stage decrease were suppressed by *kgb-1* disruption, observed in mutants carrying either the *kgb-1(um3*) allele (not shown) or the *kgb-1(km21)* allele (Fig. 5B black columns). Furthermore, presenting the mirror image to *vhp-1* knock-down, knock-down of *kgb-1* (for two generations) caused approximately a twofold increase in the prevalence of intestinal DAF-16::GFP nuclear localization in adults, from 3% in control-treated animals to 8% in *kgb-1(RNAi)* adults (*N* = 100 and 87, respectively). Together, these results support a role for KGB-1 in age-dependent antagonistic modulation of DAF-16.

To examine the contribution of *kgb-1* to modulation of endogenous DAF-16 activity, we employed a transgenic strain with *gfp* expression controlled by the promoter of the DAF-16 target gene, *sod-3*. This expression was prominent in the pharynx, intestine, and, in adults, also in the vulva. Whereas pharyngeal expression remained unchanged, intestinal expression increased in *vhp-1(RNAi-dev)* animals and decreased in *vhp-1(RNAi-ad)* animals (Fig. 5C, D); vulval expression in adults similarly decreased. Again, *kgb-1* disruption suppressed the effects of vhp-1 RNAi (Fig. 5D black columns). Together, these analyses support the notion that KGB-1 enhanced DAF-16 function in developing animals but attenuated it in adults.

### DAF-16 modulation accounts for some, but not all, of the *kgb-1*-dependent phenotypes

**Fig. 6:**
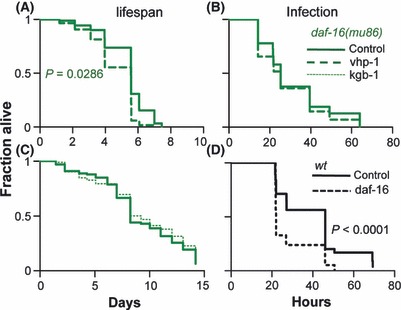
*daf-16* is necessary for some *kgb-1*-dependent detrimental phenotypes in adults. Survival curves for wild-type animals (black) and *daf-16(mu86)* mutants (green) exposed to RNAi, as designated, during early adulthood (L4 to day 2) and followed by lifespan analysis (A, C) or infection survival (B, D). When not shown, *P*-values were not significant (*P* > 0.05). Each panel shows a representative experiment of two independent experiments with similar results; each experiment in *daf-16* mutants was performed alongside a wild-type animal control (not shown) verifying that typical trends (as in Figs 1 and 4) were recapitulated.

The age-dependent contribution of *kgb-1* to both DAF-16 regulation, and stress resistance and lifespan led us to hypothesize that modulation of DAF-16 was responsible for the observed *kgb-1*-dependent phenotypes. If true, *daf-16* mutants should not be affected by KGB-1 activation or disruption. This was the case with regard to *kgb-1*’s detrimental contribution to infection resistance and lifespan in adult animals: *vhp-1* knock-down only marginally decreased the lifespan of *daf-16* animals (Fig. 6A) and did not sensitize them to infection (Fig. 6B). Furthermore, knock-down of *kgb-1* itself did not increase the lifespan of *daf-16* mutants, in contrast to its effects in wild-type animals (Fig. 6C). It should be noted that increased infection resistance following development-stage *vhp-1* knock-down, which depends on *pmk-1,* was still apparent in *daf-16* mutants (Fig. S8). Our model further predicted that *daf-16* knock-down should phenocopy *vhp-1* knock-down in adults. Previous work indeed showed that *daf-16(RNAi-ad)* animals had shortened lifespan (Dillin *et al.*, 2002). We further found that such animals also had decreased infection resistance (Fig. 6D). This is an adult-specific phenomenon, as *daf-16* disruption in larvae does not affect infection resistance (e,g, (Garsin *et al.*, 2003)).

Our results demonstrated that *daf-16* regulation underlies adult-specific detrimental effects of *kgb-1* on infection resistance and lifespan. However, we found that the contribution of *kgb-1* to cadmium resistance was independent of *daf-16,* with both vhp-1 and kgb-1 RNAi treatments having largely the same effects as in wild-type worms (Fig. S9). As MEK-1 and SHC-1, KGB-1’s activator and its scaffold protein, respectively, were previously shown to be required in the epidermis for their contribution to cadmium resistance (Mizuno *et al.*, 2008) and as our data showed *kgb-1*-dependent regulation of DAF-16 prominently in intestinal cells, it is possible that KGB-1 contributes to cadmium resistance in a *daf-16*-independent manner in the epidermis, whereas its detrimental effects on infection resistance and lifespan depend on DAF-16 modulation in intestinal cells. Support for this hypothesis was provided by experiments in *sid-1(qt2)* mutants, in which knock-down is limited to the intestine. While *vhp-1* knock-down sensitized *sid-1* adults to infection, similar to wild-type animals, it had no effect on cadmium resistance (Fig. S9). Overall, the experiments in *daf-16* mutants demonstrated the involvement of *daf-16* in *kgb-1*-dependent detrimental effects in adults. However, the contribution of intestinal *kgb-1*-dependent DAF-16 nuclear localization in developing animals remains an open question.

## Discussion

The dichotomy in the contribution of JNK signaling, stress protective vs. tissue damaging, is a fundamental feature of JNK signaling described in both mammals and drosophila. It is accepted that context has a dominant role in determining the outcome of JNK activation, but what defines this context(s) is not fully understood. Here, we show that age defines a context determining the outcome of JNK activation. We found that the *C. elegans* JNK homolog, KGB-1, protected developing larvae from heavy metals and protein folding stress but sensitized young adults to the same stressors (as well as to bacterial infection) and shortened lifespan under normal conditions. The reversal in *kgb-1*’s contribution was manifested following inactivation (*kgb-1* knock-down) and hyperactivation (*vhp-1* knock-down) alike, demonstrating that the degree of activation is secondary to the phenomenon itself and pointing at the relevance of the reversal of *kgb-1* contribution to the normal *C. elegans* physiology.

**Fig. 7:**
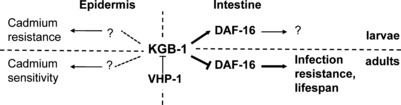
Tissue-specific and age-dependent contributions of KGB-1. A model summarizing the identified age-dependent contributions of *kgb-1*; boldface represents aspects of the mechanism that were worked out; capitals represent gene products. KGB-1 enhances nuclear localization of DAF-16 in the intestine of larvae (with yet unknown consequences) but attenuates this localization in adult animals, leading to infection susceptibility and, in the long term, shortening lifespan. For cadmium resistance, KGB-1 activation is protective in larvae but sensitizes adults. Both contributions are extra-intestinal, most likely in the epidermis (Mizuno *et al.*, 2008), but the mediator(s) are yet unknown. Not shown in the model is PMK-1, activation of which is invariably infection protective.

Underlying the reversal in *kgb-1*’s contribution, we found a reversal in the effects of KGB-1 on DAF-16’s output, suggesting that DAF-16 is a mediator of KGB-1’s age-dependent opposing contributions to stress resistance. Although *daf-16* was indeed found by epistasis analysis to be necessary for the detrimental effects of *kgb-1* on infection resistance and lifespan, it was not involved in the effects of *kgb-1* on cadmium resistance in either age; furthermore, the significance of its *kgb-1*-dependent nuclear localization in the larval intestine is still unknown (Fig. 7). Knock-down experiments targeting specifically intestinal gene expression showed that unlike infection resistance, cadmium resistance was not affected by intestinal KGB-1 activation. Together with a previous study reporting that cadmium resistance depended on epidermal-specific JNK signaling (Mizuno *et al.*, 2008), this suggests that KGB-1 activation in different tissues contributes to distinct stress phenotypes and that DAF-16 is an intestine-specific mediator, while other proteins may mediate the contribution of *kgb-1* outside of the intestine, for example in the epidermis. Lastly, how KGB-1 interacts with DAF-16 is yet unknown, but previous work focusing on the neuronal homolog JNK-1 suggests that KGB-1 may physically interact with DAF-16 (Oh *et al.*, 2005).

Our results suggest that the dichotomy in JNK signaling goes beyond tissue specificity, as opposite effects of KGB-1 activation are observed in the same tissue in different ages. Furthermore, *vhp-1* knock-down in different ages results in similar increases in phosphorylated KGB-1, suggesting that the extent of activation does not change with age. Our results are consistent with the hypothesis that age itself (onset of adulthood) determines a set-point that reverses the outcome of JNK signaling in the same tissue. They further suggest that past a certain developmental stage, the contribution of JNK signaling becomes mainly detrimental. If the trend identified in *C. elegans* extends to mammals, which is quite possible based on the known parallels, it would help to explain the detrimental involvement of JNK signaling in various age-related pathologies. Furthermore, it would suggest JNK proteins as potential targets for inhibition to ameliorate such pathologies, with minimal detrimental consequences.

The model emerging for *kgb-1*, describing a beneficial contribution during development but detrimental contribution past development, is reminiscent of the characteristics predicted by the antagonistic pleiotropy theory for the evolution of aging. This theory proposes that aging has evolved as a consequence of an age-associated decline in selection pressure, permitting genes with late-life deleterious effects to be positively selected if they confer an advantage early in life (Williams, 1957). While this theory proposes a framework to explain how traits causing aging could be selected, it does not offer concrete underlying mechanisms. Any protein/pathway that is important early in life – for development, or reproduction – and is detrimental later, as evidenced by lifespan extension following disruption in adults, may represent a mechanism of antagonistic pleiotropy. A prominent example of this is the conserved nutrient-sensing protein kinase TOR (target of rapamycin), which positively regulates mRNA translation; its disruption during *C. elegans* development causes growth arrest or lethality, but its inhibition in adults prolongs lifespan (Hansen *et al.*, 2007). Target of rapamycin is the more pivotal of several genes involved in protein synthesis that show similar trends (Chen *et al.*, 2007; Curran & Ruvkun, 2007). Whereas such observations support antagonistic pleiotropy and point to the important role played by the regulation of protein synthesis in lifespan determination, they do not explain how this occurs. In the absence of such mechanistic details, it remains unclear what would make a mechanism that is beneficial early in life become detrimental later. Two possible scenarios (of several possible ones) include a switch in protein function and tipping a balance between co-existing beneficial and detrimental contributions; neither could be evaluated without a mechanistic example of antagonistic pleiotropy. In fact, it was argued that convincing examples of antagonistic pleiotropy would be hard to find owing to the difficulty of knowing the contribution of a gene in different parts of the life cycle (Williams, 1957). However, current methods for conditional gene knock-out or age-specific knock-down enable just that. The age-dependent switch that we identified in the contribution of *kgb-1* to stress resistance, particularly in the contribution to cadmium resistance, supports a model in which a developmental program changes the beneficial contribution of a protein to detrimental.

One of the implications of the antagonistic pleiotropy theory was assumed to be the great number of pleiotropic genes contributing to aging, as any large negative contribution of a gene variant to late adult life could be offset by a relatively small advantage conferred early in life, potentially contributing to high variability in such genes among individuals. This may be taken to signify that the chance of singling out common causes of aging was very small. Our findings, however, present a case of a pleiotropic gene with a significant positive contribution early in life. Whether this is the rule or the exception for pleiotropic aging genes remains to be seen, but if the former, it may suggest that some causes of aging could be by-products of essential processes making them more likely to be shared among individuals and therefore increasing the chances of identifying targets for the treatment of aging.

Our results provide evidence that age is an important factor determining the role JNK signaling plays in contributing to stress protection and health. Furthermore, they provide tangible evidence moving the phenomenon of antagonistic pleiotropy from an abstract driving force in the evolution of aging to a mechanism that contributes to proximal features of aging and by doing so elaborates on the original model. A better understanding of KGB-1’s detrimental effects has the potential to shed more light on the evolution of senescence and on aging itself.

## Experimental procedures

**Strains** used appear in the relevant sections. A complete list appears as Supporting Information.

**RNAi by feeding** was performed at 25 °C, typically for 2 days – from egg stage to the terminal L4 stage (RNAi-dev) or from L4 to second day of adulthood (RNAi-ad). Sterilization by *cdc-25.1* knock-down was performed as described elsewhere (Shapira & Tan, 2008).

**Survival assays** were performed at 25 °C in triplicate with approximately 100 animals per group, per experiment. Animals were transferred to experimental plates immediately following RNAi. Lifespan, cadmium, and tunicamycin assays were performed on NGM plates, NGM supplemented with 100 μm CdCl_2_, or NGM supplemented with 10 μg mL^−1^ tunicamycin, respectively; in all cases, worms were fed kanamycin-killed *E. coli.* Experiments with high (5 mm) cadmium were performed in liquid K medium (53 mM NaCl, 32 mM KCl) without food. *P. aeruginosa* infection experiments were performed using the slow-killing protocol described elsewhere (Tan *et al.*, 1999).

### Development assays

Animals were grown on NGM plates containing 50 μm CdCl_2_ (at 25 °C) or 1 μg mL^−1^ tunicamycin (at 20 °C). Following 3 days, percentage of animals reaching adulthood was counted. 50–170 eggs per strain/treatment were assayed, and experiments were performed twice.

### RNA extraction and quantitative (q)RT-PCR

RNA was extracted using Trizol (Invitrogen, Grand Island, NY, USA) from 100 to 200 worms per sample and treated with Turbo DNAse (Ambion, Grand Island, NY, USA). For qRT-PCR measurements, gene-specific threshold cycle (*C*_t_) values were normalized to the respective actin values and presented as fold changes over the appropriate control samples. For a list of primers, see Supporting Information.

**Immunoblotting** was performed based on standard procedures (see Data S1 for details) using the following antibodies at the indicated dilutions: anti-pKGB-1 (Mizuno *et al.*, 2008), gratefully received from Dr. Kunihiro Matsumoto, Nagoya, Japan, 1:300; anti-pPMK-1 (9215, 1:1000; Cell Signaling Technology, Danvers, MA, USA); and anti-actin (sc-10731, 1:200; Santa Cruz Biotechnology, Santa Cruz, CA, USA). Secondary antibodies were peroxidase-conjugated donkey anti-rabbit (711-035-152, 1:2500; Jackson Immunoresearch, West Grove, PA, USA). Band intensities were measured with Photoshop and normalized to their local background, as well as to band densities in actin immunoblots.

**GFP imaging** employed *daf-16::gfp* or P_*sod-3*_::*gfp* transgenic animals. For cadmium experiments, animals were placed on NGM plates or NGM+100 μm CdCl_2_ at the L4 stage for 1 day at 25 °C. Animals were washed off, paralyzed with 25 mm levamisole (Sigma, St. Louis, MO, USA), and mounted on slides, altogether taking < 5 min, to minimize ‘slide stress’. Images were acquired using identical settings for control and experimental samples. Whole-worm GFP intensity was quantified in *P*_*sod-3*_::*gfp* animals using Metamorph (Molecular Devices, Sunnyvale, CA, USA), subtracting local background intensity for each worm. 20–30 animals were quantified per group, per experiment. Nuclear localization in DAF-16::GFP worms was measured as percentage of animals with intestinal nuclear localization. Each experiment included 30–200 animals per group.

### Statistical analyses

Differences between survival curves were evaluated using Kaplan–Meier analysis followed by logrank test or, when indicated, the Wilcoxon's test; the latter allocates more weight to early time points. Differences between one time point measurements performed in several occasions (i.e., survival on high CdCl_2_, qRT-PCR) were assessed using two-way anova with the experimental group as one factor and time of experiment as the other. Differences in DAF-16 nuclear localization were assessed using a paired *t*-test.

## Supporting Information

**Fig. S1** Changes in infection resistancefollowing *vhp-*1 knock-down are not associated with changesin reproduction.

**Fig. S2** Effects of development-stage *vhp-1* knock-down are outweighed by its effects in adults.

**Fig. S3** *kgb-1(um3)* mutants are immune tothe detrimental effects of adult *vhp-1* knock-down.

**Fig. S4** Age-dependent reversal in *kgb-1*'s contribution to tunicamycin resistance.

**Fig. S5** RNAi knock-down efficiency in larvae and adult animals is comparable.

**Fig. S6** Increases in *kgb-1*, *pmk-1* and *vhp-1* expression with age

**Fig. S7** DAF-16 nuclear localization in *vhp-1(RNAi-dev)* animals is not correlated with its effects on their development.

**Fig. S8** *daf-16* disruption does not prevent increased infection resistance in animals exposed to vhp-1 RNAi during development.

**Fig. S9** Age-dependent contribution of *kgb-1* to cadmium resistance is independent of *daf-16*.

**Data S1** Supporting experimental procedures.

As a service to our authors and readers, this journal provides supporting information supplied by the authors. Such materials are peer-reviewed and may be re-organized for online delivery, but are not copy-edited or typeset. Technical support issues arising from supporting information (other than missing files) should be addressed to the authors.
