# Supplementary material for: An age-dependent reversal in the protective capacities of JNK signaling shortens Caenorhabditis elegans lifespan
Source: Aging Cell. 2012 Aug;11(4):659–67. doi: 10.1111/j.1474-9726.2012.00829.x (PMC3440580; doi:10.1111/j.1474-9726.2012.00829.x)
Supplement: Supplementary file 1 [file acel0011-0659-SD1.pdf]

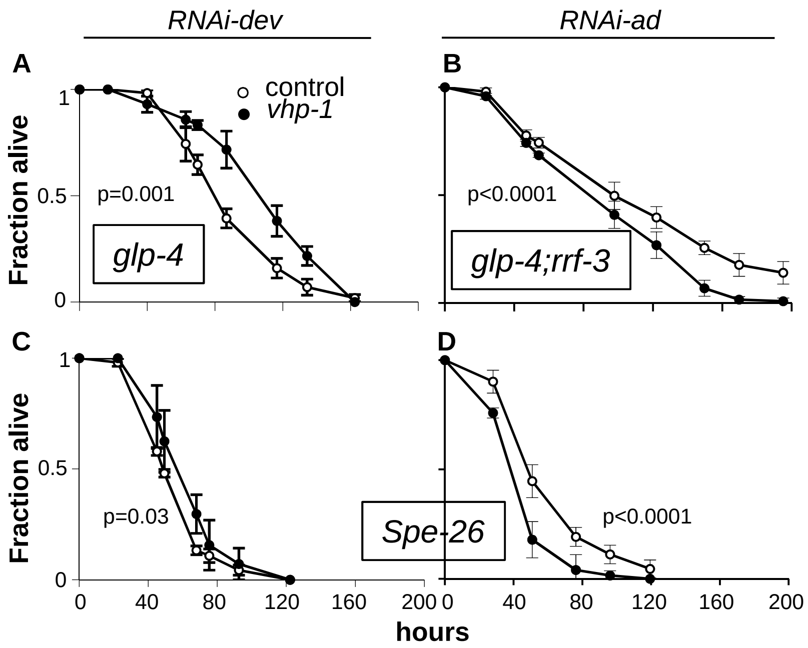

Figure S1. Changes in infection resistance following vhp-1 knock-down are not associated with changes in reproduction. Survival curves for gonad-less glp-4(bn2) mutants treated with RNAi from egg stage until L4 (RNAi-dev) (A), glp-4(bn2);rrf-3(pk1426) mutants treated with RNAi from the L4 stage for two days (RNAi-ad) (B), or sperm-defective spe-26(it112) mutants treated with RNAi from the egg (C) or L4 stage (D). In all cases, the effect of vhp-1 RNAi is the same as that obtained in wild-type fertile worms. Shown are means ± SD of fraction of live animals on each of 3 plates

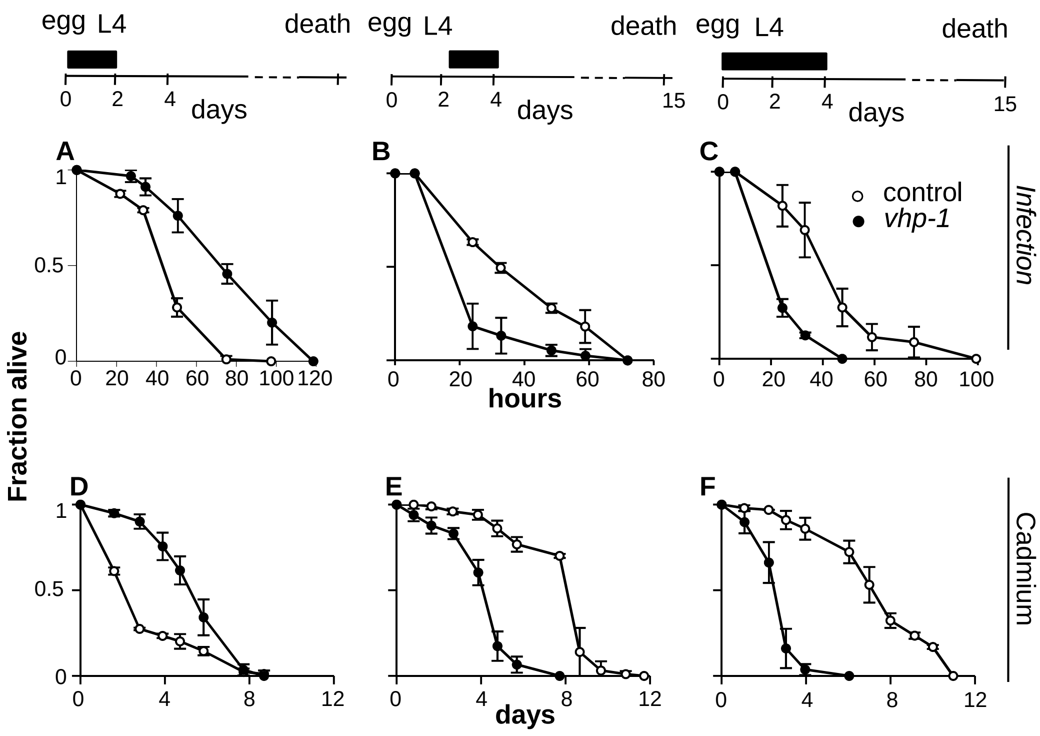

Figure S2. Effects of development-stage *vhp-1* knock-down are outweighed by its effects in adults. Survival curves for wild-type animals treated with the designated RNAi clones and infected with *P. aeruginosa* (A-C)or exposed to 100 µM cadmium chloride (D-F). Time course of RNAi treatment was as depicted in the accompanying schemes (bars). In both infection and cadmium assays, animals exposed to vhp-1 RNAi from the egg stage for 4 days develop into small adults, yet present decreased stress resistance (C and F)**,** similar to the *vhp-1(RNAi-ad)* animals shown in (B and E) and in contrast to egg RNAi for 2 days (A and D). $p<0.0001$ for all panels. This suggests dominance of the effects caused by vhp-1 RNAi in adults over those caused by development-stage knock-down. Furthermore, since the treatment in B adds the treatments of RNAi-dev and RNAi-ad, yet results in the same phenotype as in A, this phenotype is not likely to be due to decreased RNAi efficiency in adults compared to developing larvae.

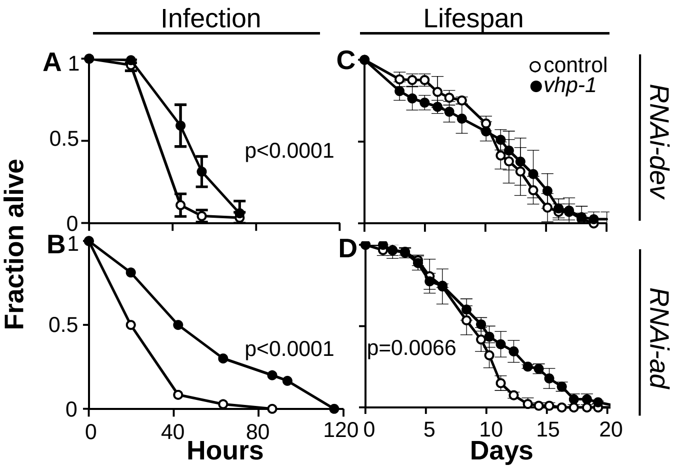

Figure S3. *kgb-1(um3)* mutants are immune to the detrimental effects of adult *vhp-1* knock down. Survival curves of *kgb-1(um3)* mutants treated with designated RNAi from the egg stage until L4 (*RNAi-dev*) or from the L4 stage for two days (*RNAi-ad*) and assayed for either survival on *P. aeruginosa* (A, B) or lifespan on dead *E.*coli (C, D).

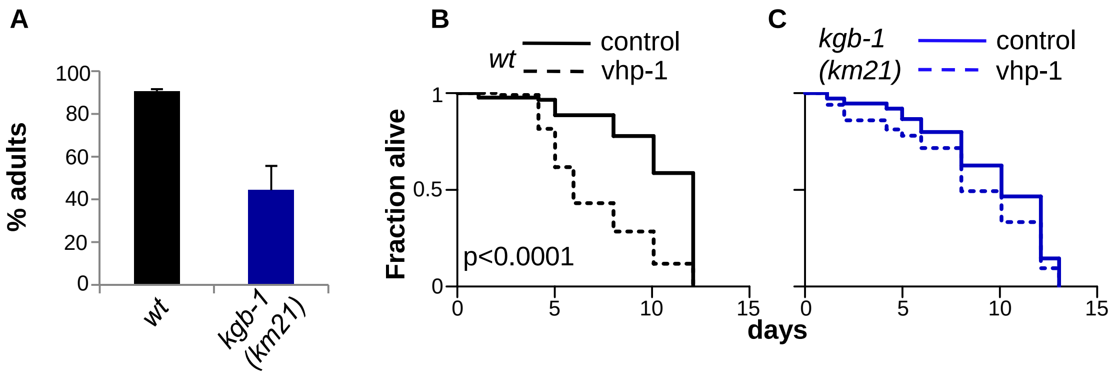

Figure S4. Age-dependent reversal in *kgb-1*'s contribution to tunicamycin resistance. (A) Percentage of animals reaching adulthood within 3 days of development on 1µg/ml tunicamycin at 20°C. Shown are averages of two independent experiments, each with >100 animals per strain. (B,C) Survival on 10µg/ml tunicamycin following vhp-1 knock-down in adults.

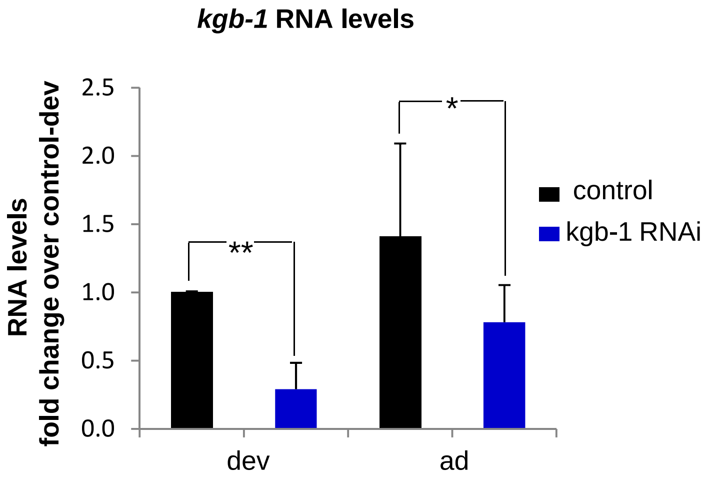

Figure S5. RNAi knock-down efficiency in larvae and adult animals is comparable. RNA levels measured by qRT-PCR in wildtype animals treated with RNAi from egg to L4 stage (dev) or from L4 to two-day old adult (ad). Shown are means and SD of data from two experiments. **$p<0.0001$, *$p=0.0018$ (2-way ANOVA for effects of experiment and treatment).

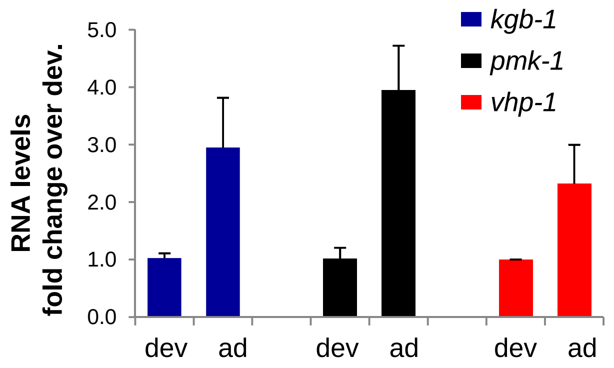

Figure S6. **Increases in *kgb-1, pmk-1* and *vhp-1* expression with age.** mRNA levels measured by qRT-PCR in wildtype L4 stage (dev) or two-day old adult (ad) animals. *vhp-1* data is for the a-isoform. Shown are means and SD of duplicate measurements from three experiments (*kgb-1 and pmk-1*) or two experiments (*vhp-1*). $p<0.001$ for each gene

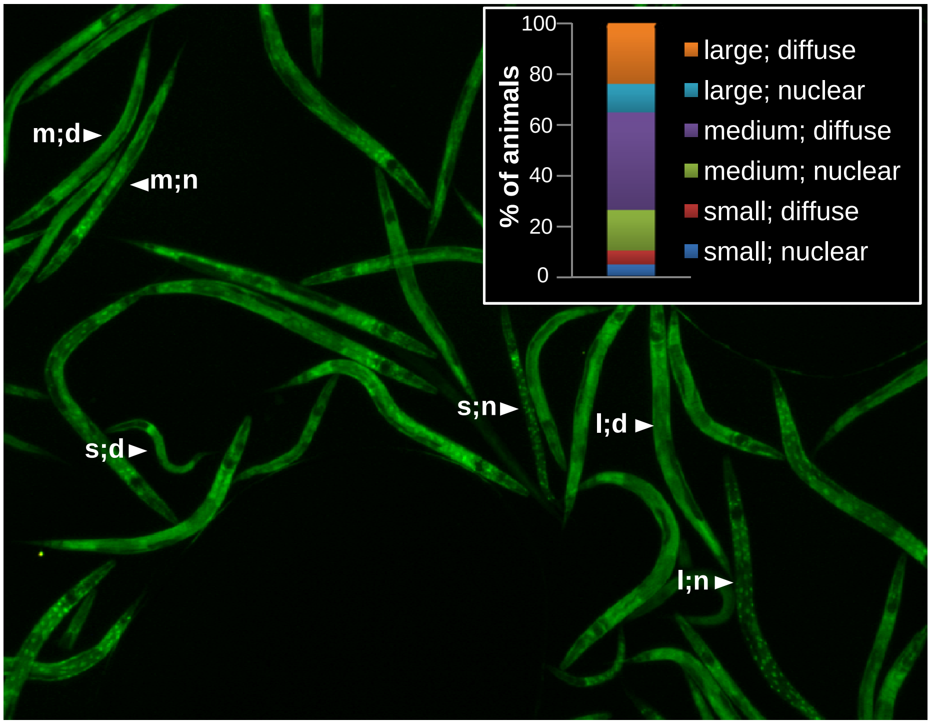

Figure S7. DAF-16 nuclear localization in *vhp-1(RNAi-dev)* animals is not correlated with its effects on their development. Representative image of DAF-16::GFP-expressing animals treated with vhp RNAi from the egg stage, showing nuclear localization in worms of different sizes/developmental stages (s, small; m, medium sized; l, large; n, nuclear; d, diffused DAF-16). **Inset,** break-down of all worms in similar images according to size and DAF-16 localization. N=1066 animals. The relative proportion of animals with nculear localization was similar in all size groups.

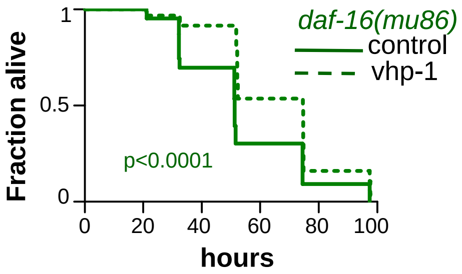

Figure S8. ***daf-16*** **disruption does not prevent increased infection resistance in animals exposed to vhp-1 RNAi during development.** Survival curve of *daf-16* mutants treated with vhp-1 RNAi during development and subsequently exposed to *P. aeruginosa*.

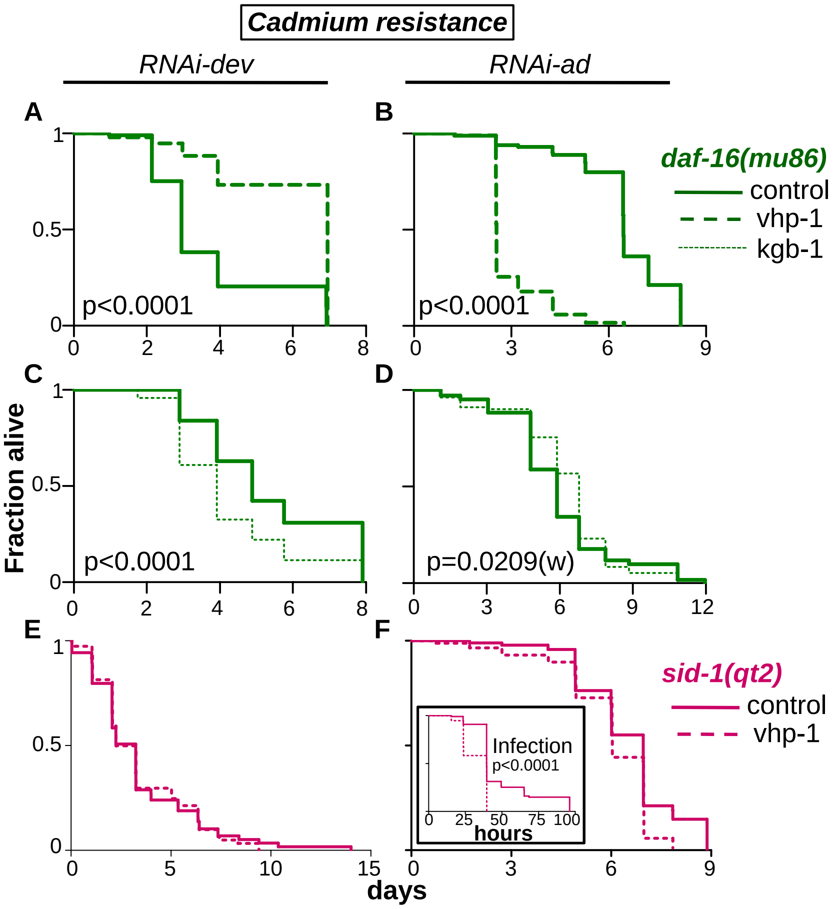

Figure S9. The age-dependent contribution of *kgb-1* to cadmium resistance is extra-intestinal and *daf-16*-independent. Survival curves for *daf-16* mutants treated with vhp-1 (A,B) or kgb-1 RNAi (C,D), in the designated stages, or *sid-1* mutants treated with vhp-1 RNAi (E,F), followed by exposure to 100µM $CdCl_2$. Inset in (F) demonstrates that while *vhp-1* knock-down in *sid-1* mutants did not affect cadmium resistance (unlike its effects on wildtype animals), it decreased infection resistance in the same worms similar to wildtype animals.
