# Supplementary material for: An age-dependent reversal in the protective capacities of JNK signaling shortens Caenorhabditis elegans lifespan
Source: Aging Cell. 2012 Aug;11(4):659–67. doi: 10.1111/j.1474-9726.2012.00829.x (PMC3440580; doi:10.1111/j.1474-9726.2012.00829.x)
Supplement: Supplementary file 2 [file acel0011-0659-SD2.doc]

**Supporting Experimental Procedures**

**Worm strains.** Worm strains were obtained from the *Caenorhabditis* Genetics Center and maintained using standard procedures. Strains included are: N2, as the wild-type strain; KU25, *pmk-1(km25);* KU21, *kgb-1(km21*), carrying a deletion spanning exons 4 and 5 of *kgb-1*, which was previously shown to suppress the larval arrest phenotype caused by *vhp-1* disruption (Mizu*no et a*l. 2004)*;* KB3, *kgb-1(um3)*, carrying a deletion spanning exons 6-9 of *kgb-1*, including the entire active site (Smi*th et a*l. 2002); SS104, *glp-4(bn2); glp-4(bn2);rrf-3(pk1426*)(Shapi*ra et a*l. 2006)*;* BA837, *spe-26(it112);* NH3119*, shc-1(ok198);* FK171*, mek-1(ks54);* CF1038, *daf-16(mu86)*; CF1553, muIs84*[P_sod3_::gfp];* TJ356, zIs356*[P_daf-16_::daf-16::gfp;rol-6]*; and HC75, *sid-1(qt2);*ccIs4251, with a disruption in the gene encoding a dsRNA channel necessary for systemic RNAi ,thus restricting knock-down to the intestine (Winst*on et a*l. 2002). Strains *kgb-1(um3)*;*P_daf-16_::daf-16::gfp* and *kgb-1(um3)*;*P_sod3_::gfp* were generated by crossing strain KB3 with TJ356 and CF1553 respectively, and strain *kgb-1(km21)*;*P_daf-16_::daf-16::gfp* was generated by crossing strain KU21 with TJ356.

**RNAi.** *vhp-1* was targeted by two RNAi clones showing similar results in infection assays. The identity of the Open Biosystems library clone, (Rebo*ul et a*l. 2003) was confirmed by sequencing and used throughout this study. All other RNAi clones were from the Ahringer library(Kama*th et a*l. 2003).

**PCR Primers.**

F08G5.6 forward CACAATGATTTCAATGCGAGA

F08G5.6 reverse GTTTCGACCGAGAAATCGAG

*lys-2* forward CCAATATCAAGCTGGCAAGG

*lys-2* reverse GTTGGATTGTTTGGCCAGTT

*kgb-1* forward TTGCGCACAAACTCTGGTAG

*kgb-1* reverse CACCGGACACTTCACTTTCA

*pmk-1* forward GCCAATGTTTCCACAGACAA

*pmk-1* reverse TCAGCACAAACAGTTCC

*vhp-1* forward TCTCGAAACTCATCAGAAGACG

*vhp-1* reverse TCCATTTTTGTGCAACCTGA

pan-actin forward TCGGTATGGGACAGAAGGAC

pan-actin reverse CATCCCAGTTGGTGACGATA

**Immunobloting**. Approximately 500-600 worms were harvested with M9 buffer and re-suspended in Radio-Immunoprecipitation Assay (RIPA) buffer containing protease and phosphatase inhibitor cocktails (Calbiochem and Roche, respectively). Homogenization was achieved using a combination of pestle homogenization, freeze-thaw cycles and sonication. The resulting homogenate was spun briefly and total protein content in the supernatant was determined using the Coomassie (Bradford) Protein Assay Kit (Pierce Biotechnology). Fifty micrograms of total protein were loaded per lane, separated on a 10% Tris-HCl polyacrylamide gel (Bio-Rad) and transferred in 20% methanol buffer at 4^o^C to Amersham Hybond-P PVDF membranes (GE Healthcare). Prior to incubation with primary antibodies, membranes were blocked with 5% BSA in TBST (for pKGB-1 antibodies, gratefully received from Dr. Kunihiro Matsumoto, Nagoya, Japan (Mizu*no et a*l. 2008), 1:300), or Western Breeze® Blocking solution (Invitrogen) (for pPMK-1 (Cell Signaling Technology, 9215, 1:1000), and actin (Santa Cruz Biotechnology, sc-10731, 1:200). Secondary antibodies were peroxidase-conjugated donkey anti-rabbit (Jackson Immunoresearch, 711-035-152, 1:2500). Signal was detected using Western Lightning ECL kit (Perkin Elmer). Band intensities were measured with Adobe Photoshop, and normalized to their local background, as well as to band densities in actin immunoblots.

**REFERENCES**

Kamath RS, Fraser AG, Dong Y, Poulin G, Durbin R, Gotta M, Kanapin A, Le Bot N, Moreno S, Sohrmann M, Welchman DP, Zipperlen P , Ahringer J (2003). Systematic functional analysis of the Caenorhabditis elegans genome using RNAi. *Nature*. 421, 231-237.

Mizuno T, Fujiki K, Sasakawa A, Hisamoto N , Matsumoto K (2008). Role of the Caenorhabditis elegans Shc adaptor protein in the c-Jun N-terminal kinase signaling pathway. *Mol Cell Biol*. 28, 7041-7049.

Mizuno T, Hisamoto N, Terada T, Kondo T, Adachi M, Nishida E, Kim DH, Ausubel FM , Matsumoto K (2004). The Caenorhabditis elegans MAPK phosphatase VHP-1 mediates a novel JNK-like signaling pathway in stress response. *Embo J*. 23, 2226-2234.

Reboul J, Vaglio P, Rual JF, Lamesch P, Martinez M, Armstrong CM, Li S, Jacotot L, Bertin N, Janky R, Moore T, Hudson JR, Jr., Hartley JL, Brasch MA, Vandenhaute J, Boulton S, Endress GA, Jenna S, Chevet E, Papasotiropoulos V, Tolias PP, Ptacek J, Snyder M, Huang R, Chance MR, Lee H, Doucette-Stamm L, Hill DE , Vidal M (2003). C. elegans ORFeome version 1.1: experimental verification of the genome annotation and resource for proteome-scale protein expression. *Nat Genet*. 34, 35-41.

Shapira M, Hamlin BJ, Rong J, Chen K, Ronen M , Tan MW (2006). A conserved role for a GATA transcription factor in regulating epithelial innate immune responses. *Proc Natl Acad Sci U S A*. 103, 14086-14091.

Smith P, Leung-Chiu WM, Montgomery R, Orsborn A, Kuznicki K, Gressman-Coberly E, Mutapcic L , Bennett K (2002). The GLH proteins, Caenorhabditis elegans P granule components, associate with CSN-5 and KGB-1, proteins necessary for fertility, and with ZYX-1, a predicted cytoskeletal protein. *Dev Biol*. 251, 333-347.

Winston WM, Molodowitch C , Hunter CP (2002). Systemic RNAi in C. elegans requires the putative transmembrane protein SID-1. *Science*. 295, 2456-2459.
